# Retraction: Transdermal Delivery of Insulin by Amidated Pectin Hydrogel Matrix Patch in Streptozotocin-Induced Diabetic Rats: Effects on Some Selected Metabolic Parameters

**DOI:** 10.1371/journal.pone.0247150

**Published:** 2021-02-11

**Authors:** The PLOS ONE Editors

Following the publication of this article [1], concerns were raised regarding results presented in Figs 1 and 6. Specifically,

- Fig. 1B partially overlaps with Fig. 1D.
- In Fig. 6, the following bands appear similar:
  ○ Left β-actin panel: lanes 1 and 8, and lane 5 when flipped.
  ○ Left β-actin panel: lanes 2 and 6 when flipped.
  ○ Left β-actin panel: lanes 3 and 4, and lane 7 when flipped.
  ○ Right β-actin panel: lanes 1 and 3.
- In the Fig. 6 GS panel, left β-actin panel and right β-actin panel irregularities have been detected between the background signal directly surrounding the bands in all lanes and the overall background signal of the blot.

The original data for the figures of concern are no longer available.

In light of the concerns affecting multiple figure panels that question the integrity of these data, the *PLOS ONE* Editors retract this article.

SIH, PSN, and MRS either did not respond directly or could not be reached. CTM is deceased.
